# An endoscopic approach to therapy for spontaneous esophageal rupture

**DOI:** 10.1016/j.vgie.2022.05.001

**Published:** 2022-07-09

**Authors:** Kristin Lescalleet, Tala Mahmoud, Sudhir Duvuru, Andrew C. Storm

**Affiliations:** Division of Gastroenterology and Hepatology, Mayo Clinic, Rochester, Minnesota

**Keywords:** EOE, eosinophilic esophagitis, NJ, nasojejunal

## Abstract

Video

## Background

Boerhaave syndrome is among the most lethal GI tract disorders with a reported mortality rate of up to 40%.^1^, ^2^, ^3^ Spontaneous perforations are caused by sudden intraesophageal pressure elevations leading to transmural injury and subsequent mediastinal inflammation, subcutaneous emphysema, and/or necrosis secondary to spillage of gastric contents. Management was historically surgical, but stable patients may benefit from the reduced morbidity and mortality associated with less invasive percutaneous and endoscopic approaches. Our case demonstrates one technique for successful management of Boerhaave syndrome with an emphasis on teaching and troubleshooting.

## Case

**Figure 1:**
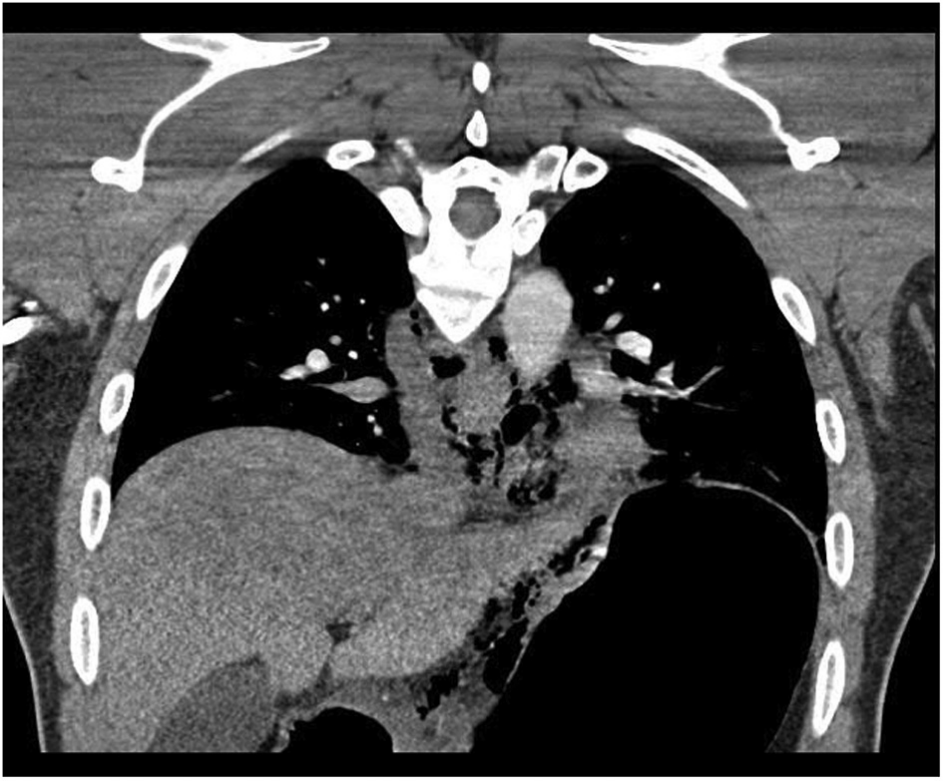
Chest CT scan showing a complex heterogenous air and fluid-filled mediastinal collection.

**Figure 2:**
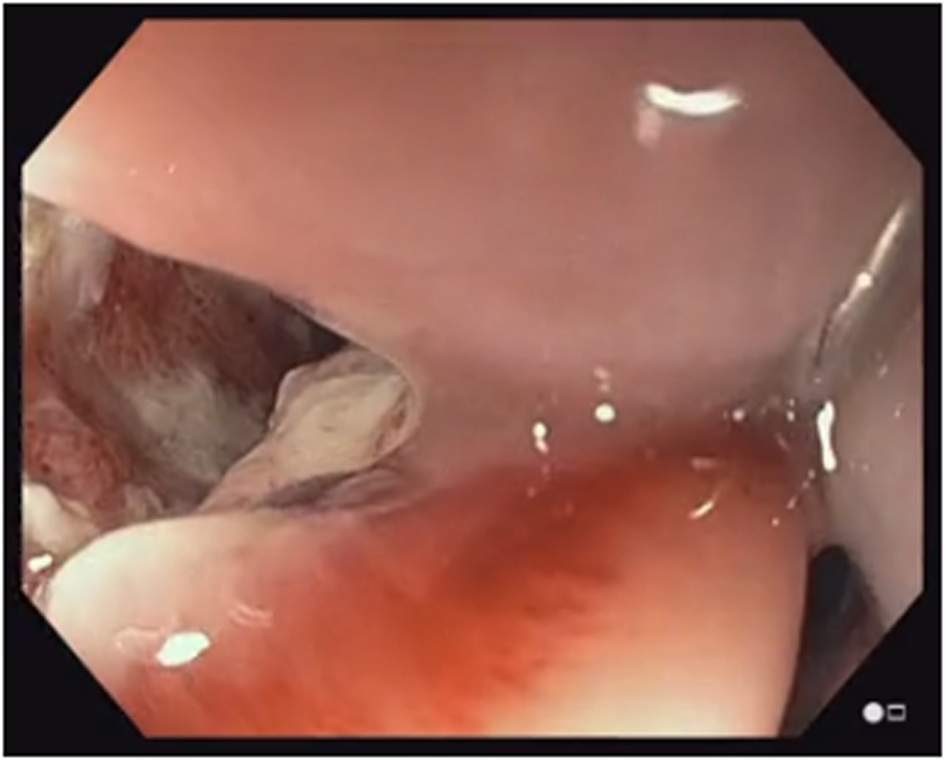
Endoscopic image depicting the gastroesophageal junction (right) and the site of esophageal perforation (left).

**Figure 3:**
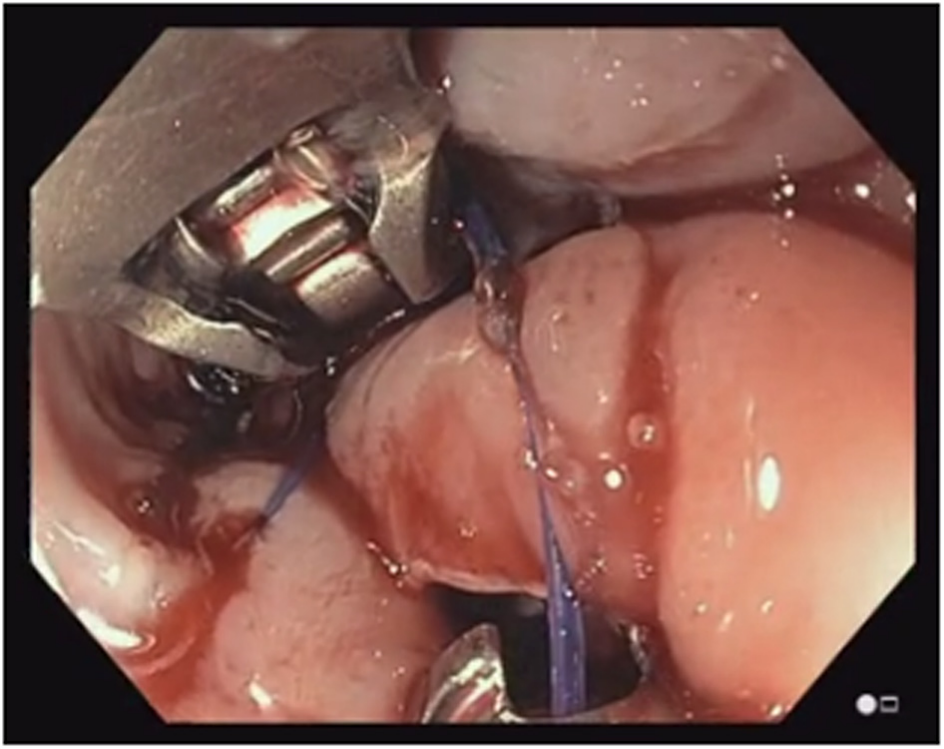
Endoscopic suturing for closure of the perforation (bottom) moving from distal to proximal site of perforation in a running suture pattern.

**Figure 4:**
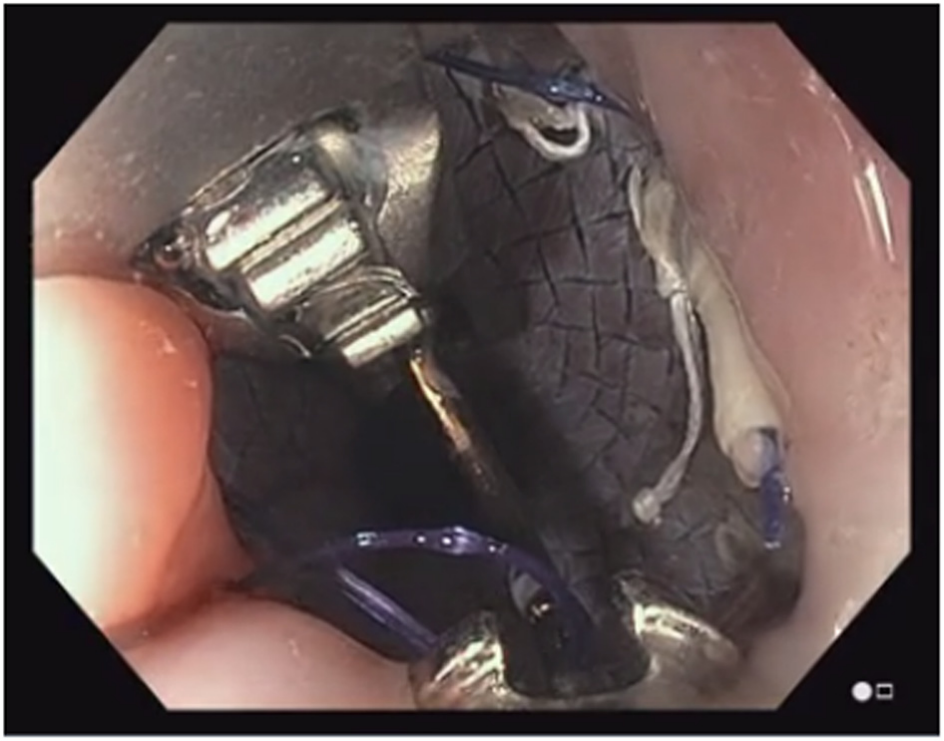
Placement of stay sutures through the interstices of the stent. Pictured are the T-tag from the first stay suture placement (3 o’clock position) and mid-suture placement of the second stay suture placement.

**Figure 5:**
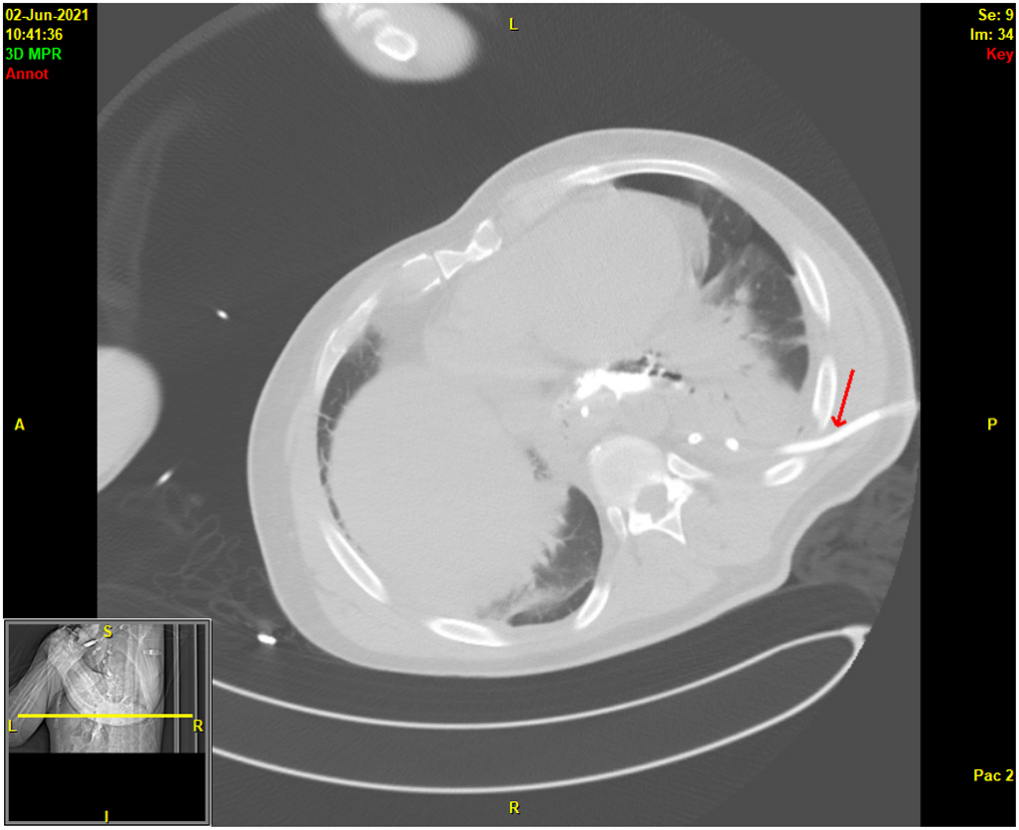
Chest CT scan showing placement of a percutaneous drain for the mediastinal air and fluid-filled collection.

**Figure 6:**
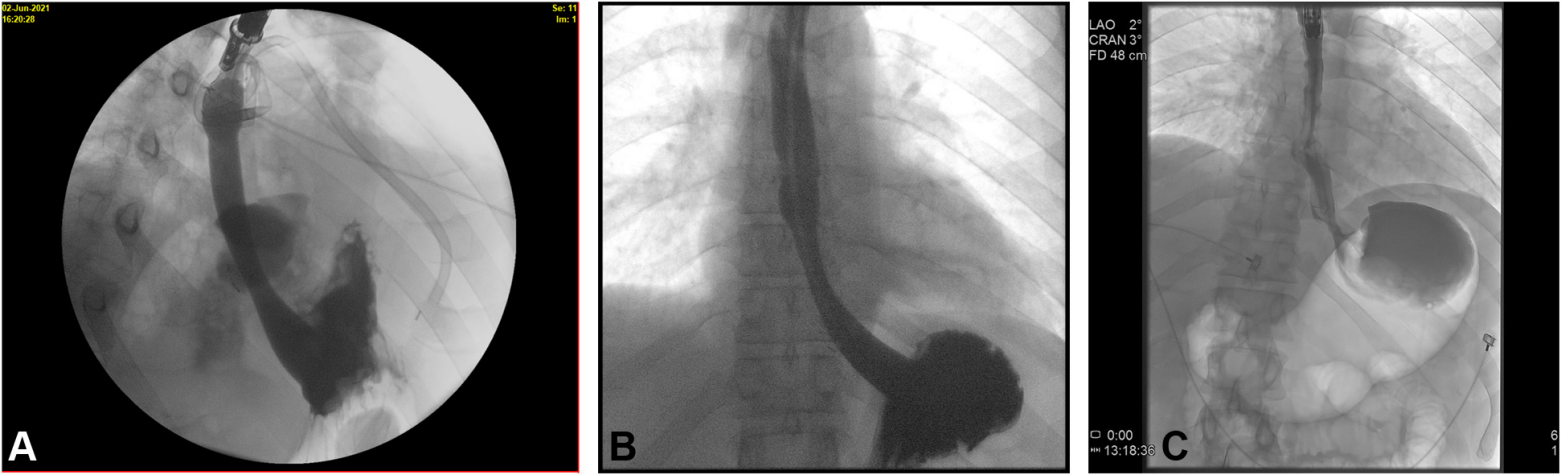
Progression of healing of rupture through upper GI series. **A,** Post–stent placement. **B,** Fourteen days post–stent placement. **C,** Post–stent removal showing complete resolution in the esophageal rupture.

**Figure 7:**
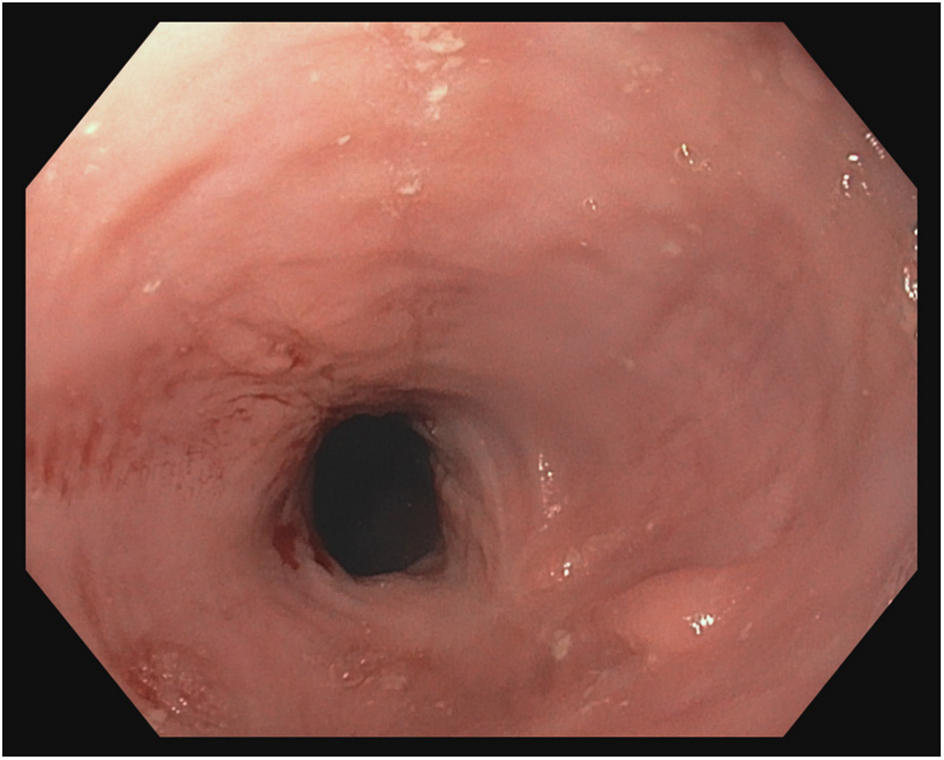
Follow-up endoscopy 6 months after stent removal showing complete healing of the distal esophagus.

A 38-year-old otherwise healthy man presented with substernal chest pain after self-induced vomiting while attempting to resolve a food impaction (Video 1). A chest CT scan showed a complex heterogenous air and fluid-filled mediastinal collection suggestive of esophageal rupture (Fig. 1). Given his hemodynamic stability, the patient was immediately referred for urgent endoscopic evaluation and therapy. The patient was intubated and sedated for endoscopic closure approximately 8 hours after esophageal rupture (Fig. 2). During the procedure, the patient’s hemodynamics and ventilation were monitored by critical care anesthesia and used minimal amounts of carbon dioxide insufflation with intermittent endoscopic suction to decrease the risk of capnothorax and capnomediastinum. As with any extramural endoscopic procedure, the endoscopist was prepared to provide needle decompression to treat any cardiopulmonary decompensation. The perforation was repaired via endoscopic suturing (Apollo OverStitch; Austin, Tex, USA) (Fig. 3) with overlapping, fully covered, through-the-scope stent placement (TaeWoong Niti-S; Cook Medical, Winston-Salem, NC, USA) and stay sutures (Fig. 4). A nasojejunal (NJ) feeding tube was placed for enteral feeding beyond the perforation. After endoscopic closure, the mediastinal fluid collection with tracking into the pleural cavity was drained via chest tube placement (Fig. 5). The patient underwent weekly upper GI series to monitor stent position, patency, and lack of contrast extravasation (Fig. 6). After 14 days, the chest tube was removed because of no ongoing output, and at 21 days, the patient underwent a repeat upper endoscopy with stent removal and biopsies to rule out EOE. At the patient’s 6-month follow-up, he was doing well and remained asymptomatic on a once-daily proton pump inhibitor (Fig. 7).

## Procedural Steps

1. Multidisciplinary consultation with thoracic surgery and/or interventional radiology regarding need for percutaneous drainage and/or operative interventions.
2. General anesthesia for endoscopic examination.
3. Primary full-thickness suture closure of the perforation with care to avoid extraluminal placement of T-tag or cinch and avoid overtightening the sutures or cinch, which may result in tissue ischemia and failure of the repair.
4. Overlapping covered esophageal stent placement, with stay sutures to prevent migration.
5. Nothing by mouth with NJ tube nutrition.
6. Weekly upper GI series to assess stent position, patency, and protection of the perforation site.
7. Endoscopic stent retrieval between 2 and 4 weeks, depending on clinical progress. If fully covered, the stent may be moved fully to the stomach, the repair site checked for closure, and then the stent either replaced across the repair or removed depending on healing.
8. Rule out and treat medical etiologies for perforation including eosinophilic esophagitis (EOE) and GERD.

## Limitations of the Technique

1. Excess capnothorax or capnomediastinum should be carefully avoided (use minimal insufflation, use suction, and use irrigation) to prevent cardiopulmonary adverse events.
2. Esophageal rupture spills “dirty” gastric contents into the mediastinum, and depending on quantity and severity, may often require percutaneous drainage of this developing phlegmon or effusion if the primary repair and stent are to succeed.
3. Stent placement prevents direct visualization of the sutured repair during the course of healing.
4. Stent placement (radial force) may compromise integrity of the repair, so choose the stent diameter carefully.
5. Stent removal may traumatize the repair site or other areas of the esophagus. Careful removal and/or consideration of using an “inversion technique” for removal is advised.
6. A purely endoscopic repair for all cases is not advised. Careful multidisciplinary approach including anesthesia, cardiothoracic surgery, and interventional radiology, where available, will improve safety and outcomes.
7. Close follow-up is needed. When endoscopic approaches fail, percutaneous or surgical intervention should be promptly sought.

## Conclusions

Primary endoscopic repair with endoscopic suturing and overlapping stent placement and stay sutures to prevent stent migration is a viable option for management of spontaneous esophageal perforation in an otherwise stable patient, as shown in this case. If a large pleural or mediastinal collection is present, drainage is required, often with percutaneous chest tube or drain placement. Working in a multidisciplinary approach is required for optimal patient outcomes.

## Disclosure

*Dr Storm has received research grants from Apollo Endosurgery, Boston Scientific, Endogenex, Endo-TAGSS, and Enterasense and is a consultant for Apollo Endosurgery, GI Dynamics (data safety board), ERBE (data safety board), Olympus, and Intuitive Surgical. All other authors disclosed no financial relationships.*
